# Oxygen Generating Biomaterials Preserve Skeletal Muscle Homeostasis under Hypoxic and Ischemic Conditions

**DOI:** 10.1371/journal.pone.0072485

**Published:** 2013-08-26

**Authors:** Catherine L. Ward, Benjamin T. Corona, James J. Yoo, Benjamin S. Harrison, George J. Christ

**Affiliations:** Wake Forest Institute for Regenerative Medicine, Wake Forest University School of Medicine, Winston-Salem, North Carolina, United States of America; University of Minnesota Medical School, United States of America

## Abstract

Provision of supplemental oxygen to maintain soft tissue viability acutely following trauma in which vascularization has been compromised would be beneficial for limb and tissue salvage. For this application, an oxygen generating biomaterial that may be injected directly into the soft tissue could provide an unprecedented treatment in the acute trauma setting. The purpose of the current investigation was to determine if sodium percarbonate (SPO), an oxygen generating biomaterial, is capable of maintaining resting skeletal muscle homeostasis under otherwise hypoxic conditions. In the current studies, a biologically and physiologically compatible range of SPO (1–2 mg/mL) was shown to: 1) improve the maintenance of contractility and attenuate the accumulation of HIF1α, depletion of intramuscular glycogen, and oxidative stress (lipid peroxidation) that occurred following ∼30 minutes of hypoxia in primarily resting (duty cycle = 0.2 s train/120 s contraction interval <0.002) rat extensor digitorum longus (EDL) muscles *in vitro* (95% N_2_–5% CO_2_, 37°C)*;* 2) attenuate elevations of rat EDL muscle resting tension that occurred during contractile fatigue testing (3 bouts of 25 100 Hz tetanic contractions; duty cycle = 0.2 s/2 s = 0.1) under oxygenated conditions *in vitro* (95% O_2_–5% CO_2_, 37°C); and 3) improve the maintenance of contractility (*in vivo*) and prevent glycogen depletion in rat tibialis anterior (TA) muscle in a hindlimb ischemia model (i.e., ligation of the iliac artery). Additionally, injection of a commercially available lipid oxygen-carrying compound or the components (sodium bicarbonate and hydrogen peroxide) of 1 mg/mL SPO did not improve EDL muscle contractility under hypoxic conditions *in vitro*. Collectively, these findings demonstrate that a biological and physiological concentration of SPO (1–2 mg/mL) injected directly into rat skeletal muscle (EDL or TA muscles) can partially preserve resting skeletal muscle homeostasis under hypoxic conditions.

## Introduction

The provision of supplemental oxygen to hypoxic skeletal muscle following trauma-related ischemia remains a major medical challenge with important implications for tissue salvage, repair and regeneration. Clinically, prolonged periods of ischemia can lead to skeletal muscle death and gross tissue necrosis [1], for which there currently are limited treatments. In an effort to prevent ischemia-related tissue death, several general strategies have been explored for provision of oxygen to various tissues under a variety of ischemic and/or hypoxic conditions.

For example, hyperbaric oxygen therapy [2], which super-saturates blood plasma with oxygen, has been shown to improve wound healing and limb salvage following vascular-related trauma [3], [4]. Similarly, compounds that increase the oxygen carrying capacity of the blood and other physiological solutions have been studied. Hemoglobin- and perfluorocarbon-based oxygen carriers (HBOCs and PFCs) [5] have been tested as blood substitutes and have yielded varying degrees of success in improving outcomes following tissue hypoxia, yet, they are also associated with some distinct disadvantages related to clearance from the body and unfavorable side effects such as flu-like symptoms and esophageal dysfunction. Lipid-based oxygen carriers [6], [7], [8], [9] have been tested for organ preservation and have shown extended times for sustained metabolic activity of tissues contained within *in vitro* organ bath systems. However, these oxygen-carrying technologies are dependent on the presence of an intact vascular system for distribution of oxygen: a scenario that is not always present, as in conditions of compartment syndrome, trauma or disease [1], [10], [11], [12]. In addition, technologies to increase vascularization [13] that may be damaged or removed during such circumstances have also been explored and have displayed accelerated vessel growth, though with limited efficiency and slow development.

A novel class of oxygen-*generating* biomaterials represents a viable solution for the direct provision of oxygen to hypoxic skeletal muscle in the event of compromised blood flow. Specifically, sodium percarbonate (SPO) is a fast-releasing oxygen compound that appears promising for tissue salvage applications. SPO is an adduct of sodium carbonate and hydrogen peroxide that, in the presence of water, readily decomposes into oxygen, water, and other salts. Previously, SPO has been used in commercial applications as a rich source of oxygen, such as in soil remediation to improve bacteria viability [14], as well as a cleaning agent for teeth [15], [16]. In addition, this material has also been used successfully as a therapeutic agent for skin wound healing in a mouse skin flap model by delaying the onset of necrosis up to 3 days and decreasing cellular apoptosis [17]. However, oxygen-generating compounds, including SPO, have not been tested specifically for skeletal muscle applications.

Skeletal muscle has a broad and plastic metabolic capacity designed to efficiently support the energy requirements of the tissue. Although glycolytic metabolism may also contribute, energy is primarily generated by oxidative metabolism at rest and during activity (i.e., exercise) in muscles comprised of predominantly slow oxidative and fast glycolytic fibers [18], [19], [20]. In response to hypoxia (or anoxia), ATP concentrations are maintained acutely (∼4 hr; [1], [21], [22]), but are eventually diminished resulting in tissue necrosis [1]. Acutely, hypoxia is associated with an increased presence of free radicals that diminish muscle contractility in a manner independent of high-energy phosphate depletion [22], [23], [24], [25]. To this end, administration of antioxidants to hypoxic tissue has been shown to attenuate reductions in contractile force [22], [25], indicating that contractility is a sensitive global index of acute hypoxia-driven alterations in cellular redox homeostasis.

The purpose of the current investigation was to determine if SPO could support skeletal muscle metabolism under hypoxic conditions, using the loss of contractility as a primary index of loss of metabolic homeostasis [25], [26]. To do so, we report a series of studies that encompass characterizing the SPO biomaterial in a cell-free environment (*Study One*) to testing identified biologically and physiologically compatible concentrations of SPO in skeletal muscle under hypoxic or ischemic conditions *in vitro* (*Study Two*) *and in vivo* (*Study Three*), respectively. Collectively, the findings of these studies demonstrate that SPO (1 mg/mL) represents a biocompatible oxygen-generating material capable of partially supporting skeletal muscle homeostasis under otherwise hypoxic/ischemic conditions.

## Methods

### Experimental Design

A series of three studies was performed to establish SPO as an effective treatment for hypoxic skeletal muscle. In *Study One,* SPO biomaterial characteristics (e.g., pH changes, free radical production, and oxygen generation) were determined in a physiological cell-free system. In *Study Two*, SPO biocompatibility and its ability to maintain skeletal muscle homeostasis under hypoxic conditions *in vitro* was determined. To assess biocompatibility, C_2_C_12_ cell viability and isolated rat extensor digitorum longus (EDL) muscle contractility assays were performed across a range of SPO concentrations under oxygen-saturated conditions (ambient air or 95% O_2_, 5% CO_2_, respectively). Upon determining a biocompatible SPO concentration (1 mg/mL) for this model, the capacity of SPO to maintain EDL muscle homeostasis *in vitro* under resting hypoxic conditions and during an active fatigue protocol was determined. Finally, in *Study Three,* SPO was tested as a tissue salvage treatment *in vivo* in a rat model of hindlimb ischemia.

### Ethics Statement

All animal-related protocols were approved by the Animal Care and Use Committee of Wake Forest University and carried out with strict adherence to the guidelines set forth. All surgeries were performed under appropriate anesthesia with postoperative pain medication.

### Chemicals

All chemicals were received from Sigma Aldrich, unless otherwise stated. Lifor® preservation medium was purchased from the company’s website (Lifeblood Medical, Inc., New Jersey, USA) and stored at 4°C, according to manufacturer’s instructions.

### Theoretical Determination of a Therapeutic Concentration of SPO

The goal of this study was to supply enough oxygen via SPO decomposition to at least maintain resting skeletal muscle metabolism at 37°C. Oxygen consumption for rat EDL muscle has previously been reported to be ≈0.06 µL/g/s at rest at 23–37°C [18], [19]. For the EDL muscles used in this study, which weigh, on average, approximately 130 mg, it is assumed that during the course of a 30-minute organ bath contractile protocol (described below) with a duty cycle approximating 0, the muscle will consume approximately 14.0 µL of oxygen in a resting state. The following equations were used to determine the concentration of SPO required to produce a comparable volume of oxygen:

Using the ideal gas law (PV = *n*RT), the moles, *n*, of oxygen at P = 1 atm and T = 37°C is:
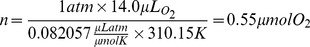
(1)


SPO decomposes to generate oxygen according to the following equation:
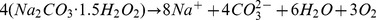
(2)


The amount of SPO needed to generate the theoretical value for oxygen required by the resting rat EDL is:
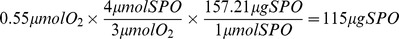
(3)


The volume of solution administered to the tissue *in vitro* was selected as 20 µL: a volume that did not diminish *in vitro* force production (Figure S1). Concentrations of SPO solutions tested either in cell culture or *in vitro* contractility experiments included 0.001, 0.01, 0.1, 1, 5, and 10 mg/mL to determine toxicity, physiological compatibility and efficacy of SPO. Isolated EDL contractility experimental muscles received a total of 0.002, 0.02, 0.1, and 0.2 mg SPO– a range that encompassed the requirement of oxygen to maintain resting metabolism under these conditions (*Eq. 3*).

### Study I: Characterization of SPO Biomaterial in a Cell-free System

#### Scanning electron microscopy imaging

Sodium percarbonate (SPO) was cryogenically ground (Freezer/Mill®, SPEX SamplePrep) for 30 minutes and sifted through a 25 µm mesh sieve to obtain a homogenous distribution of small particles. Scanning electron microscopy (SEM; Model S-2260N, Hitachi Co. Ltd., Japan) was performed to observe the particle size and confirm uniform diameters.

#### Oxygen, pH and hydrogen peroxide measurements

SPO was added to a physiologically relevant solution of Dulbecco’s modified Eagle’s medium (DMEM, Gibco®) at 0, 1 and 10 mg/mL, and oxygen generation, pH changes, and hydrogen peroxide production were measured (n = 3). An Oxygen Biosensor System (OBS, 96-well plate, Becton Dickinson™) was used to determine oxygen concentration (mg/L) per the manufacturer’s guidelines. Briefly, samples in triplicate were placed in the wells, and the OBS plate was kept in a hypoxic glovebox system (Biospherix, Ltd.) set to ∼0% O_2_, 5% CO_2_. Fluorescence intensity of the OBS plate was read on a fluorometer (Molecular Devices, Spectramax M5; 485 nm excitation, 630 nm emission). Readings were correlated to fluorescence intensities of known dissolved oxygen concentrations of DMEM solutions obtained by incubating solutions under calibrated oxygen settings in incubators. pH measurements of solutions in 15 mL conical tubes were made using a pH probe (Accumet®, Fisher). Hydrogen peroxide content was quantified using an Amplex® Red Hydrogen Peroxide/Peroxidase Kit (Invitrogen®) [27]. Fluorescence was measured using a fluorometer (545 nm excitation, 590 nm emission). Oxygen, pH and peroxide measurements were taken periodically, up to 24 hours.

### Study II: *In vitro* Analysis of SPO

#### C2C12 viability assay

Viability studies were performed to test cytocompatibility of SPO at concentrations later used in tissue studies. Murine myoblasts (C_2_C_12_, passage 5) were cultured in complete growth medium (DMEM containing 10% fetal bovine serum, 100 U/mL penicillin and 100 µg/mL streptomycin), plated at 3,000 cells/well in a 96-well tissue culture-treated plate and allowed to adhere for 24 hours in a 37°C humidified atmosphere of 20% O_2_ – 5% CO_2_. After attachment SPO at concentrations of 0, 0.001, 0.01, 0.1, 1 and 10 mg/mL (n = 8/[SPO]in growth medium) was applied to the cells. Additionally, bovine catalase (100 U/mL), which accelerates the decomposition of hydrogen peroxide to water and oxygen and acts as an antioxidant [28], was added to half of the wells at each [SPO]. After 2 hours of exposure to experimental treatments under normal incubation (20% O_2_ − 5% CO_2_, 37°C), a 3-(4,5-dimethylthiazol-2-yl)-2,5-diphenyl-tetrazolium bromide (MTT) assay was performed to assess metabolic function of the cells. In brief, cells were washed with phosphate buffered saline (PBS), MTT reagent was added to the wells for 4 hours (5 mg MTT/mL media), and the blue formazan precipitate was extracted from the mitochondria using dimethyl sulfoxide (DMSO). Absorbance of the solution was read using a spectrophotometer (Spectramax M5; 540 nm). Quantification of a viable cell population was expressed as the ratio of absorbance of the growth control (only cells).

#### *In vitro* EDL muscle functional testing

Six month old female Lewis rats (∼200 g) were anesthetized using isoflurane. Extensor digitorum longus (EDL) muscles (average weight of 130.9±1.8 mg) were isolated for *in vitro* functional assessment as previously described [29]. Immediately after isolation, EDL muscles were placed in ice-cold Krebs-Ringer bicarbonate buffer (pH 7.4) with (in mM) 121.0 NaCl, 5.0 KCl, 0.5 MgCl_2_, 1.8 CaCl_2_, 24.0 NaHCO_3_, 0.4 NaH_2_PO_4_, and 5.5 glucose, at which time they were treated as per their experimental grouping (Table 1). When applicable, treatments were administered via two intramuscular injections (30G needle) tracking along the long-axis of the muscle and a volume of 10 µL was administered with each injection, starting at the midbelly and tracking to the proximal or distal end of the muscle.

**Table 1:** Treatment groups for *in vitro* hypoxic skeletal muscle contractility experiments.

Name	Treatment
Untreated	No treatment
Needle	Sham injection only receiving two needle sticks on each end (bilateral) of the muscle
Saline	Vehicle control receiving two injections of saline only
SPO 0.1 mg/mL	Experimental group receiving two injections of 0.1 mg/mL concentration of SPO biomaterial in saline vehicle
SPO 1.0 mg/mL	Experimental group receiving two injections of 1.0 mg/mL concentration of SPO biomaterial in saline vehicle
SPO 5 mg/mL	Experimental group receiving two injections of 5 mg/mL concentration of SPO biomaterial in saline vehicle
SPO 10 mg/mL	Experimental group receiving two injections of 10 mg/mL concentration of SPO biomaterial in saline vehicle
Sodium Carbonate	Component control group receiving two injections of sodium carbonate at concentration that is present in 1 mg/mL of SPO biomaterial in saline vehicle (0.675 mg/mL)
Hydrogen Peroxide	Component control group receiving two injections of hydrogen peroxide at concentration that is present in 1 mg/mL of SPO biomaterial in saline vehicle (0.3248 mg/mL)
LOC	Commercial control group receiving two injections Lifor® organ preservation solution

After the injections (or a time interval of ∼2 minutes), muscles were mounted in a DMT organ bath system (DMT Model 750TOBS) containing a Krebs-Ringer bicarbonate buffer equilibrated with 95% O_2_ – 5% CO_2_ gas (37°C). The distal tendon was attached by silk suture and cyanoacrylate adhesive to a fixed support, and the proximal tendon was attached to the lever arm of a force transducer (DMT 750TOBS). The muscle was positioned between custom-made platinum electrodes. Following mounting, the muscles were equilibrated for 5 minutes prior to determining optimal physiological muscle length (average L_o_ of 3.4 cm) via a series of twitch contractions. Direct muscle electrical stimulation (200 ms train, 0.2 ms pulse with supramaximal voltage) was applied across the EDL muscle using a Grass S88 stimulator (Grass Instruments, Quincy, MA). Real time display and recording of all force measurements were performed on a computer with Power Lab/8sp (ADInstruments, Colorado Springs, CO).

All *in vitro* tissue responses were expressed as force (N) normalized to physiological cross-sectional area (PCSA, cm^2^), which was calculated using the following equation:
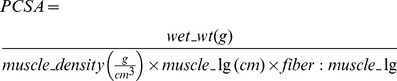
(4)


Muscle density is 1.06 g/cm^3^ and the EDL fiber to muscle length ratio is 0.44 [30], [31], [32]. Unless otherwise indicated, active specific forces are reported (peak force – resting tension). Tissues were removed from the organ bath and flash-frozen for protein and histological analyses.

#### Testing of contractility during acute hypoxia of resting muscle

Under oxygenated conditions (95% O_2_ – 5% CO_2_), isometric tetanic force as a function of the stimulation frequency (1, 60, 100 and 200 Hz, 200 ms train of 0.2 ms pulses) was measured at 37°C with 2 minutes between contractions. The gas was then changed to a 95% N_2_-5% CO_2_ mixture to create a hypoxic (<1% dissolved or <0.5 g/mL oxygen measured) environment, as previously reported [25]. The muscles were incubated under hypoxic conditions for 20 minutes; during this time, muscles were stimulated at 60 Hz every 2 minutes to monitor contractile activity. The magnitude of contractile forces were similar between treated and untreated groups during the twenty minute protocol and therefore both groups presumably consumed a similar amount of energy [20]. After this incubation, the muscle was again stimulated via a range of frequencies (60, 100 and 200 Hz) while maintained under hypoxic conditions, with the entire protocol lasting approximately 30 minutes. Because the duty cycle of the contractions during the protocol approximated zero (train duration/contraction interval; 0.2 s/120 s≈ 0.002), and therefore did not presumably induce active fatigue [33], deterioration in contractility over the protocol was primarily attributed to hypoxia related contractile dysfunction within the muscle.

#### Testing of contractility during an active fatigue protocol

The capacity of SPO to maintain skeletal muscle contractility was further assessed under oxygenated conditions but with a fatiguing duty cycle (train duration/contraction interval; 0.2 s/2 s = 0.1). Rat EDL muscle contractility can be preserved for ∼30 minutes in isolated preparations at physiological temperatures when the contractile duty cycle approximates zero [29], [33], [34]. However, as activity increases, and in suite the metabolic demand of the tissue, oxygen diffusion gradients in isolated muscle can be created, wherein even with 95% O_2_ perfused in the organ bath a hypoxic core within the muscle develops [33], [34], [35]. In this experiment, muscles underwent a similar pre-test, as described above, and then performed three bouts of 25 tetani (100 Hz, 200 ms train of 0.2 ms pulses, 2 s inter-train period, 0.1 duty cycle, 37°C) with 10 minutes rest between bouts. The active fatigue bout was estimated to result in a critical O_2_ diffusion radius of 0.2 mm [33], resulting in a 0.0674 cm^3^ hypoxic core within the EDL (considering the EDL as a cylinder with a 1 mm radius, a length of ∼3.3 cm, a mass of 130 mg, and a density of 1.06 g/cm^3^). To provide O_2_ to the entire hypoxic core (.0674 cm^3^×1.06 g/cm^3^ = 71.4 mg) assuming an oxygen consumption of 1.06 µL/s/g, as estimated from rat *in vivo* triceps surae contractile studies [20], a single bout required ∼ 3.78 µL of O_2_. Thus, with the delivery of ∼2.6 µL of O_2_ via SPO (1 mg/mL in 20 µL), we hypothesized a delay in the loss of contractility under these conditions. Tau (time to lose ∼63% of maximal active force) was calculated for each fatigue bout. To do so, contractions prior the peak measured force were removed (no significant differences between groups in the number of contractions to peak force was observed). Forces measured from the peak to the final tetani were fit with a single-decay exponential function:
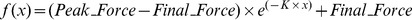
(5)


Where *x* is time (s) and *K* is the exponential rate constant. Tau is calculated as 1/*K*.

#### Comparison to alternative technology

Additional EDL muscle *in vitro* experiments were performed to compare SPO to an oxygen-carrying media designed for tissue salvage. Lifor® is a lipid-based oxygen carrier (LOC) for organ perfusion systems [6], [7], [8], [9], [36]. EDL muscles were injected with Lifor® and underwent contractile testing as described above for SPO-injected muscle.

### Study III: *In vivo* Analysis of SPO

#### In vivo ischemia injury and SPO application

After observing that SPO injection partially preserved muscle contractility in *Study Two*, SPO was further tested *in vivo* in a rat model of partial hindlimb ischemia. Anesthetized rats were aseptically prepared for surgery by sterilizing the abdomen. A longitudinal incision was made along the abdomen. The thoracic aorta was uncovered, reaching the bifurcation to the left iliac artery. The artery, vein and nerve were isolated and the iliac artery and vein were separately ligated, using 5.0 silk suture, immediately distal to the thoracic bifurcation (i.e., proximal to any subsequent bifurcations). Animals recovered for 24 hours, at which time the TA muscle was treated with one of two treatment groups: 1) saline (vehicle) injections, and 2) SPO injections. Solutions were warmed to body temperature immediately before injection. In line with the boundary conditions for SPO treatment identified in Study Two, the TA muscle was injected with 40 µL of 2 mg/mL of SPO (4 injections of 10 µL each). Care was taken to position the injections directly into the TA muscle, with the four injections evenly spaced in four quadrants of the muscle. Assuming a resting rate of oxygen consumption of 0.06 µL/s/g [18], [19], this volume of O_2_ represents ∼22% of the O_2_ requirement of the TA muscle (425 mg for 30 minutes) while remaining within biocompatible limits defined in Study Two.

Immediately after injection, isometric torque produced by the left anterior crural muscles was measured *in vivo* using similar methodology as previously described [29], [37]. After rats were anesthetized (2–2.5% isoflurane), the left hindlimb was aseptically prepared. The rat was then placed on a heated platform with an additional heat lamp to maintain physiological temperatures (∼37°C). The left knee was clamped for stability and the left foot was secured to a custom-made foot-plate that was attached to the shaft of an Aurora Scientific 305C-LR-FP servomotor, which in turn was controlled using a computer interface. Sterilized percutaneous needle electrodes were inserted through the skin for stimulation of the left common peroneal nerve. Electrical stimulus was applied using a Grass S88 stimulator with a constant current SIU (Grass Model PSIU6). Stimulation voltage and needle electrode placement were optimized first with a series of twitch contractions at 1 Hz and then with 5 isometric contractions (200 ms train of 0.05–0.1 ms pulses at 100 Hz). Then, tetanic isometric torque (100 Hz) of the anterior crural muscles was assessed every 5 minutes for a total of 30 minutes. Although the duty cycle for this protocol approximated zero (0.2 s train/300 s contraction interval <0.001), contractile fatigue was observed after iliac vessel ligation, a phenomenon similar to that previously observed [38]. For real-time analysis of torque, voltage outputs were sampled at 4000 Hz, converted to a digital signal using an A/D board (National Instruments PCI 6221) and recorded using a computer loaded with a custom-made Labview®-based program (provided by the U.S. Army Institute of Surgical Research). Functional results were expressed as a ratio of the resultant torque of the initial maximal stimulation. Hindlimb muscles were immediately removed, weighed and prepared for histological analysis.

#### Histological analyses

After *in vitro* organ bath and *in vivo* functional procedures, tissues were flash frozen in liquid nitrogen in a compound of embedding solution (OCT, Tissue-Tek®) and talcum powder and stored for histological analysis. Transverse cross-sections (9 µm in thickness) of a minimum of 3 samples per group were taken from approximately one quarter of the longitudinal length of the EDL and TA muscles to the belly of the muscle. Uninjured, native tissue that had not been exposed to the organ bath protocol was also analyzed as a proper positive control. Multiple images were taken using a Leica® upright microscope at 4x and 20x magnifications.

Immunohistochemistry was performed for hypoxia-inducible factor 1α (HIF1α). Sections were fixed in 10% formalin and endogenous peroxidase activity was blocked with Dual Endogenous Enzyme Block (Dako) at room temperature, followed by incubation with Serum-Free Protein Block (Dako). Samples were incubated in the primary antibody (HIF1α, Abcam®, ab1, mouse monoclonal, 1∶200 dilution) for 1.5 hours, followed by incubation in an appropriate secondary antibody (anti-mouse IgG (H +L), Vector®, BA-9200, 1∶200 dilution). Staining was completed with an ABC Elite kit (Vector®) and 7–8 minute incubation with ImmPact NovaRed (Vector®). Sections were counterstained with hematoxylin. Quantitative analysis was performed on six random field images (20x) per tissue by calculating the percentage of nuclei colocalized with HIF1α (appearing red-brown) compared to total nuclei per image using Image J (NIH) software.

Glycogen staining was performed by fixing sections in 10% formalin and oxidizing in 0.5% periodic acid solution for five minutes. After rinsing, sections were placed in Schiff reagent for fifteen minutes. All analyzed sections were stained simultaneously for consistency during quantification. Sections were rinsed, counterstained with hematoxylin and dehydrated for viewing. Using Image J software, three total area cross-sectional images (4x) per muscle were converted to an 8-bit image. Intensities were recorded at a 200 pt threshold, which represented the area of the section, and a 130 pt intensity, which was deemed to represent the area positive for glycogen. Ratios were calculated as the amount of glycogen-stained area per total area of the section.

#### Thiobarbituric Acid Reactive Substances (TBARS) Assay

Approximately one-third of each EDL muscle was flash frozen in liquid nitrogen for protein and lipid peroxidation analysis. A thiobarbituric acid reactive substances (TBARS) assay (Caymen Chemical) was performed on a minimum of three muscles per group, which quantifies the amount of malondialdehyde (MDA), a naturally occurring product of lipid peroxidation. Tissues were weighed and homogenized in a lysis buffer over ice. After centrifugation, the supernatant was analyzed for protein content using a Bradford assay. The TBARS assay was performed by combining sample, sodium dodecyl sulfate (SDS) and a coloring reagent which is intensified by the amount of MDA present in the sample. The samples were read using a fluorometer (530 nm excitation, 550 nm emission). All samples were normalized to specific protein concentrations and expressed as a ratio.

#### Statistical analysis

Statistical assessments were performed on functional and histological data using GraphPad Prism and SPSS software. All results were presented as the arithmetic mean ± standard error of the mean (SEM). Analysis of variance (ANOVA) was performed for all parameters. When an ANOVA revealed significance (p<0.05), a Fisher’s post-hoc test was performed.

## Results

### Study I: Characterization of Biomaterial in a Cell-free System

**Figure 1:**
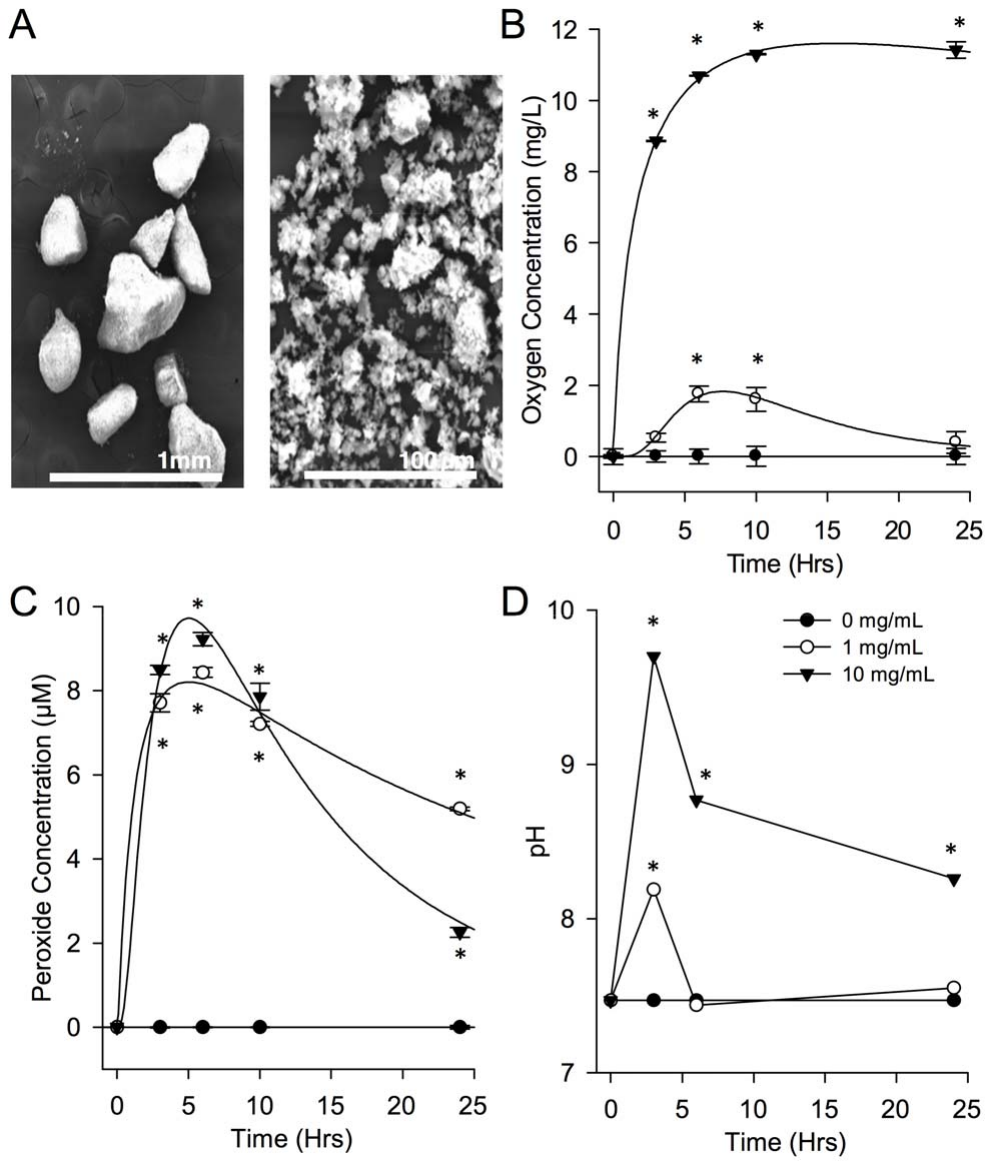
Characterization of SPO chemical decomposition in an aqueous environment. (A) SEM images of raw SPO particles before (left panel) and after (right panel) modifications to obtain small, uniform particles (<25 µm in diameter). (B) Oxygen production (mg/L) of SPO under anoxic (∼0% O_2,_ 5% CO_2_) conditions using an oxygen biosensor system (Becton Dickinson™) (n = 3). (C) Peroxide content of SPO solutions quantified over time using an Amplex Red® Assay (n = 3). (D) pH kinetics of SPO (n = 3). * Values are different from 0 mg/mL (blank) concentrations (p<0.001). Values are means ± sem.

Sodium percarbonate as received contains large particles making it incompatible for intramuscular injection (Figure 1A). Using cryogenic grinding and sifting, small homogenous particles were obtained, which exhibited diameters <25 µm (Figure 1A). These smaller particles readily dissolved and could be injected through a syringe needle. Absolute dissolved oxygen increased rapidly up to ∼10 mg/L at the highest studied concentration (10 mg/mL), which is slightly greater than the saturation of water but has been observed in hydrogen peroxide solutions [39]. After reaching a peak in the lower concentration (1 mg/mL), the oxygen concentration decreased over time, suggesting that the oxygen generation is short-lived (<3hours using this formation) and oxygen diffuses into the surrounding atmosphere (Figure 1B). Addition of SPO to physiological solutions caused an alkaline shift in the pH (Figure 1D), resulting from the carbonate ions released by SPO. pH returned to baseline values due to carbon dioxide gas exchange within the hypoxic incubators. Peroxide content of SPO samples was elevated within 3 hours of the reaction and peaked within 6 hours at approximately 10 µM, followed by a decrease towards baseline values within 24 hours (Figure 1C). These values were below toxic thresholds of peroxide observed in culture, which is approximately 10^−3^M [15]. At 24 hours, the hydrogen peroxide concentration was actually less for the 10 mg/mL than 1 mg/mL SPO group. Since hydrogen peroxide is less stable in more alkaline environments, the lower level of hydrogen peroxide detected is likely from the higher concentration of carbonate ions present which are able to create a more alkaline microenvironment. These findings suggest that hydrogen peroxide is a transient species instead of a stable byproduct of the reaction.

### Study II: *In vitro* Analysis in Hypoxic Skeletal Muscle System

#### Cell biocompatibility

**Figure 2:**
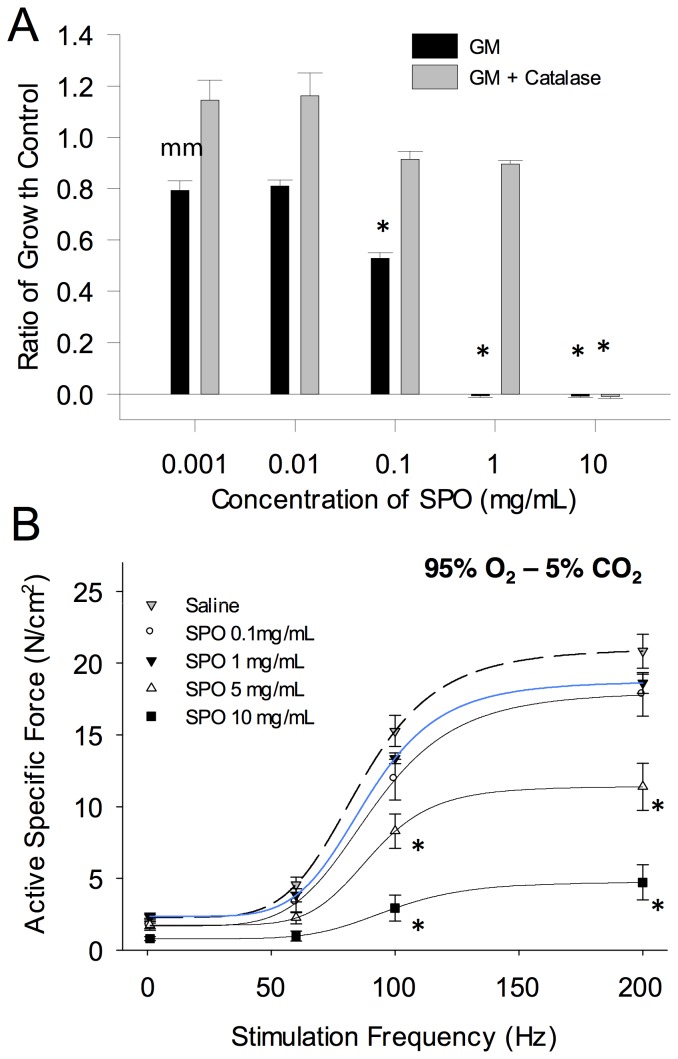
Biological and physiological compatibility of SPO. (A) MTT viability assay of C_2_C_12_ myoblasts in the presence of increasing concentrations of SPO with and without the presence of bovine catalase (100 U/mL). Assays were performed after 2 hours of cell exposure to SPO and expressed as a ratio of growth controls (no SPO content). * Values are significantly different from 0.001 mg/mL within a given media (p<0.0001, n = 8 per group). (B) Effect of saline (dashed line) and SPO (solid lines, all concentrations) on EDL muscle contractility in an oxygenated (95% O_2_–5% CO_2_) environment. * Values are different from saline group within the denoted frequency (p<0.05). Values are means ± sem; sample sizes are listed in Table 2.

To identify a biocompatible range of SPO for skeletal muscle applications, C_2_C_12_ myoblasts were incubated for two hours with SPO concentrations of 0.001–10 mg/mL. The metabolically active population of cells decreased (Figure 2A; *p<0.0001) in a SPO concentration dependent manner, wherein at low concentrations (0.001–0.1 mg/mL) cell viability was reduced by ∼20–40%, while at higher concentrations (1 & 10 mg/mL) nearly 100% cell death occurred. This was likely due to hydrogen peroxide production, as addition of the antioxidant catalase (100 U/L) completely removed the cytotoxic effects of 1 but not 10 mg/mL of SPO (Figure 2A; *p<0.0001).

#### Whole muscle biological and physiological compatibility

The effect of SPO on contractility of isolated whole EDL muscle was determined in an oxygenated (95% O_2_ – 5% CO_2_) environment (Figure 2B). The effect of the delivery method (i.e., injection via needle) on EDL force production is demonstrated in Figure S1A. Compared to saline injected control muscles, injection of 0.1 or 1.0 mg/mL SPO did not deleteriously affect force production. However, SPO concentrations of 5 and 10 mg/mL SPO reduced force at higher (100–200 Hz; *p<0.0001 compared to saline injected muscles) but not lower (1–60 Hz) stimulation frequencies (Figure 2B). Moreover, injection of the individual components of SPO (sodium carbonate and hydrogen peroxide) at concentrations found in 1 mg/mL SPO did not alter force production compared to saline injected or SPO injected muscles (Figure S1B). Collectively, these findings indicate that SPO at concentrations of 1 mg/mL or less are biocompatible, both in terms of cell viability and whole muscle contractility, when antioxidants are present.

#### Whole muscle at rest in a hypoxic environment

**Figure 3:**
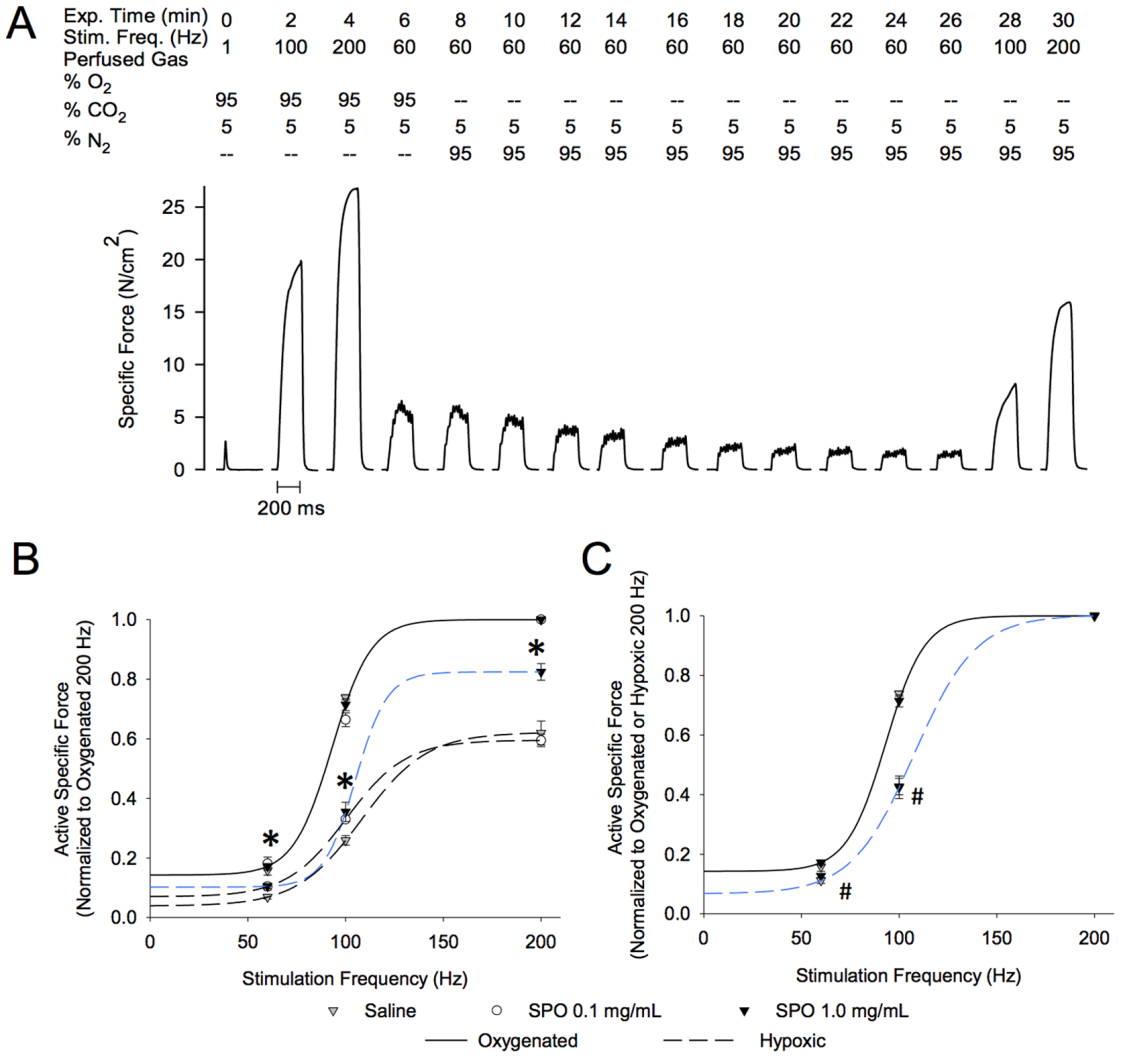
*In vitro* contractility of resting EDL muscles under hypoxic conditions with and without SPO. (A) Force tracings of a representative untreated muscle during the hypoxic protocol are depicted. (B) Per muscle, forces measured under oxygenated conditions and at the end of the hypoxic period (60, 100, and 200 Hz) were normalized to the maximal force (200 Hz) measured under oxygenated conditions. * SPO 1 mg/mL is greater than saline at each stimulation frequency. (C) Additionally, per muscle, these forces were normalized to the maximal force measured during each respective gas condition. ^#^ SPO 1 mg/mL and Saline are significantly reduced after ∼20 minutes of hypoxia (p<0.05). Values are mean ± sem; sample sizes are listed in Table 2.

Next, we tested the ability of SPO to maintain skeletal muscle contractility under hypoxic conditions (See Figure 3A for experimental design). In response to hypoxia (95% N_2_- 5% CO_2_), saline injected muscle contractility was diminished by ∼55±6, 65±3 and 38±4% at 60, 100, and 200 Hz, respectively. The greater force deficit at lower, than higher frequencies after hypoxia resulted in a downward, rightward shift in the abbreviated force-frequency curve (Figure 3B). Following the hypoxic protocol, return to oxygenated conditions (95% O_2_ – 5% CO_2_) in a subset of control muscles restored ∼15% of maximal tetanic force (Figure S2), similar to previously reported findings [40]. SPO injection of 1.0 mg/mL but not 0.1 mg/mL significantly improved the maintenance of oxygenated, pre-hypoxic force at all stimulation frequencies (Figure 3B; *p = 0.083 at 60 Hz, *p = 0.0314 at 100 Hz, *p = 0.0005 at 200 Hz, compared to saline injected muscles) –1 mg/mL SPO injected muscles had a 38±4, 50±5, & 18±3% force deficit at 60, 100, and 200 Hz, respectively. SPO (1 mg/mL) appeared to improve the downward shift of the force-frequency curve, although a rightward shift was still evident (Figure 3C; #p = 0.0189 at 60 Hz, #p = 0.0001 at 100 Hz for saline injected muscles; #p = 0.004 at 60 Hz, #p = 0.0001 at 100 Hz for SPO injected muscles). The SPO mediated maintenance of contractility was likely due to the catalytic decomposition of SPO forming O_2_, as muscles injected with the individual components of SPO (i.e., 1 mg/mL equivalents of sodium carbonate and hydrogen peroxide) did not improve the maintenance of maximal tetanic force compared to saline injected muscle (Figure S3; *p = 0.0048).

**Figure 4:**
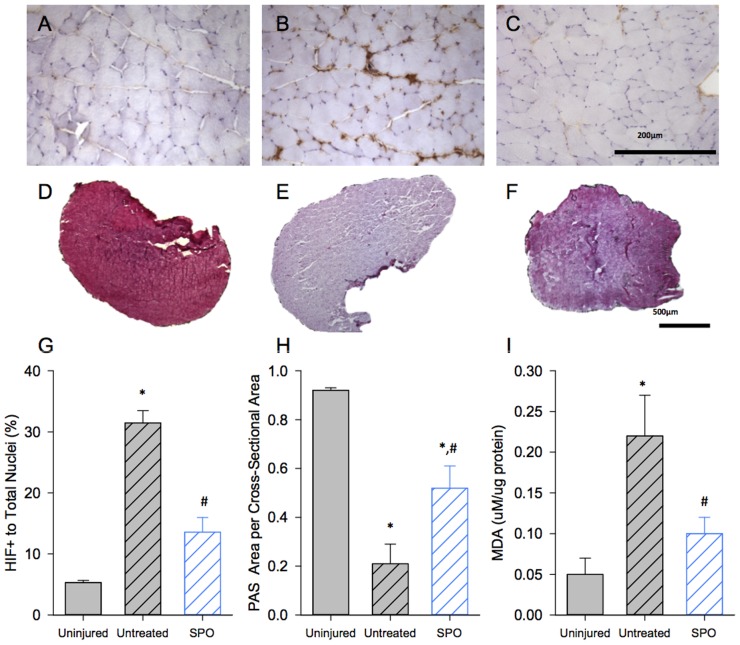
Hypoxia-induced HIF1α accumulation, glycogen depletion, and oxidative stress with and without SPO. Histological assessment of EDL muscles with HIF1α (A, B, C) and PAS glycogen (D, E, F) staining for uninjured (native) (A,D), untreated (B,E) and SPO (1 mg/mL) (C,F). Quantitative analysis was performed with HIF1α (G) and intramuscular glycogen (PAS; H) stained sections. Lipid peroxidation was assessed by quantifying MDA concentrations (I). * p<0.05 compared to uninjured; # p<0.05 compared to untreated. Values are means ± sem; sample size = 4–9/group.

Histological and biochemical metabolic indices of hypoxia further corroborated the contractile findings, that 1 mg/mL SPO decomposition generated a volume of oxygen capable of partially maintaining skeletal muscle homeostasis under hypoxic conditions. In response to hypoxia, SPO injection partially preserved intramuscular glycogen stores (*p = 0.0103 compared to uninjured muscle), which were nearly completely depleted in untreated muscles (*p = 0.0001 compared to uninjured muscle) (Figure 4 D–F, H; #p = 0.0477 between untreated and SPO injected muscles), suggesting a greater use of oxidative metabolism in coordination with SPO decomposition [1], [41]. Additionally, 1 mg/mL SPO injection significantly attenuated the increased presence of nuclear co-localized HIF1α (p = 0.1480 compared to uninjured muscle), which was observed in untreated muscles following hypoxia (*p = 0.001 compared to untreated muscle) (Figure 4 A–C, G; #p = 0.0007 between untreated and SPO injected muscles). And lastly, free radical oxidative stress or lipid peroxidation (MDA muscle content) observed in hypoxic untreated muscle (*p = 0.0229 compared to uninjured muscle) was attenuated following 1 mg/mL SPO injection (p = 0.1256 compared to uninjured muscle) (Figure 4I; #p = 0.0253 between untreated and SPO injected muscles). Collectively, these findings indicate that 1 mg/mL SPO partially prevented a hypoxia-induced metabolic sequela, and correspondingly preserved a greater level of contractility.

#### Whole muscle during activity in an oxygenated environment

**Figure 5:**
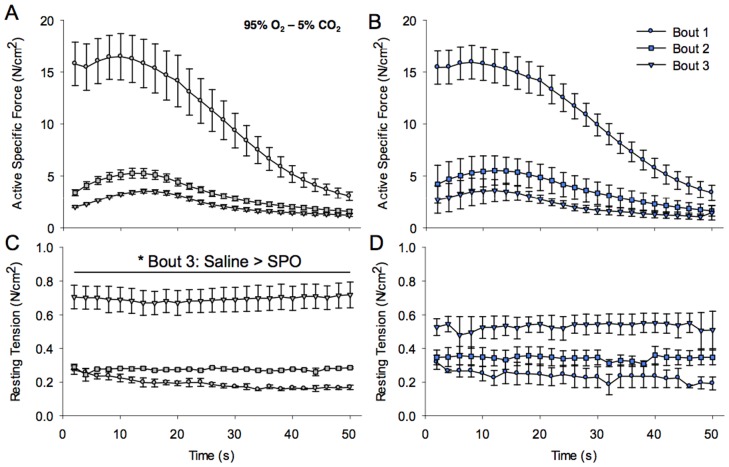
The effects of SPO on EDL muscle *in vitro* contractility during activity under oxygenated conditions. Active specific force (N/cm^2^) of saline injected (A) and SPO (1 mg/mL) injected (B) muscles was measured during three fatigue bouts of 25 tetanic contractions (100 Hz pulse frequency, 0.2 ms pulse width, 200 ms train) under oxygenated conditions (95% O_2_–5% CO_2_; 37 °C). Resting tension (N/cm^2^) of saline injected (C) muscles was significantly greater than SPO injected (D) muscle during the third fatigue bout (*p<0.05). Values are means ± sem; sample size = 3/group.

Whole rat EDL muscles were also made to perform an active fatigue protocol in an oxygenated environment (95% O_2_–5% CO_2_) with or without SPO (1 mg/mL) injection. The premise of these experiments was that this level of activity at 37°C would induce the development of a hypoxic core (*see methods;* [33], [34], [35]), which could conceivably be attenuated by SPO injection. In response to the contractile protocol, both saline- and SPO-injected muscles exhibited similar and significant active contractile fatigue profiles (Figures 5A & 5B, respectively). For example, during each successive fatigue bout, Tau was similar between experimental groups (Saline vs. SPO, n = 3/group: *Bout 1*, 13.4±0.2 vs. 16.1±2.8 s, p = 0.40; *Bout 2*, 11.9±0.4 vs. 14.4±2.5 s, p = 0.38; *Bout 3*, 8.8±0.6 vs. 8.0±1.5 s, p = 0.66). There were, however, significant differences between experimental groups in the rise in resting tension. While both groups had a similar resting tension during the first two fatigue bouts, throughout the third fatigue bout, resting tension was significantly greater for the saline than the SPO injected group (Figure 5C & 5D).

#### Oxygen-carrying vs. Oxygen-generating biomaterials

**Figure 6:**
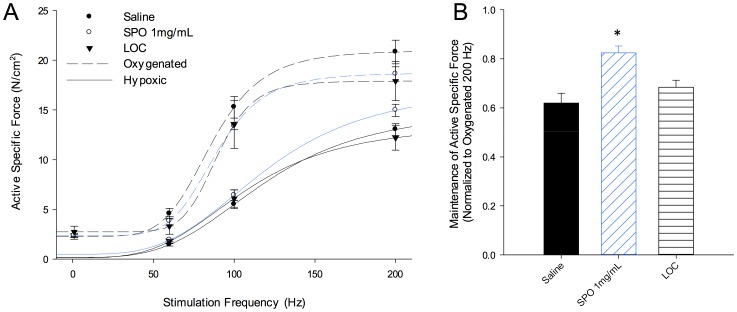
Comparison of an oxygen-generating (SPO) and an oxygen-carrying biomaterial for the preservation of EDL muscle homeostasis under hypoxic conditions *in vitro*. (A) Active specific force (N/cm^2^) generated under oxygenated (dashed lines; 95% O_2_ – 5% CO_2_) conditions and then after ∼20 minutes (*see experimental timeline in Fig. 3)* under hypoxic conditions (solid lines; 95% N_2_–5% CO_2_) by EDL muscles injected with saline, SPO (1 mg/mL) and a lipid oxygen carrier (LOC). (B) Maintenance of oxygenated maximal isometric force (200 Hz) under hypoxic conditions was significantly greater for SPO 1.0 mg/mL than all other groups, * p<0.05. Values are means ± sem; sample sizes are listed in Table 2.

To demonstrate the utility of SPO-generated oxygen for the maintenance of muscle contractility under hypoxic conditions (95% N_2_–5% CO_2_), an oxygen-carrying technology designed for similar applications was tested for comparison. The lipid oxygen carrier (LOC) system (Lifor®) chosen has previously been shown to preserve renal tissue and cardiac muscle function following hypoxic conditions [6], [7], [8], [9], [36]. However, when injected into skeletal muscle the LOC system maintained maximal force (200 Hz) at a magnitude similar to saline injected control muscles (p = 0.2037) and lesser than 1 mg/mL SPO (*p = 0.0115) (Figure 6A–B, Table 2). A variety of experiments were conducted using the LOC as organ bath solution (*as opposed to an injectable)*, which more closely approximates previously described methods of use for this LOC with other tissues [6], [7], [8], [9], [36], however, an improvement in the maintenance of force compared to saline injected muscle was not observed (*data not shown*). These findings lend further support that it is the generation of oxygen with SPO decomposition in a physiological environment that promotes the improved maintenance in contractility under hypoxic conditions.

**Table 2:** *In vitro* EDL muscle contractility under oxygenated and hypoxic conditions.

Treatment	Sample Size	Oxygenated P_o_ (N/cm^2^)	Hypoxic P_o_ (N/cm^2^)	Ratio of Maintained P_o_
Untreated	4	29.4±2.6	18.7±2.4	0.63±0.03
Needle Injection	5	20.0±1.1	13.4±0.8	0.67±0.02
Saline Injection	5	21.1±0.5	13.0±0.6	0.62±0.04
SPO Low (0.1 mg/mL)	4	17.8±1.5	10.6±1.2	0.59±0.02
SPO Mid (1.0 mg/mL)	10	18.3±0.7	15.0±0.6	0.83±0.03*
Sodium Carbonate	4	18.1±1.0	9.7±1.4	0.54±0.07
Hydrogen Peroxide	6	19.3±1.1	11.9±1.4	0.62±0.07
LOC	5	17.9±2.0	12.2±1.3	0.68±0.03

Values (mean ± sem) are active maximal isometric specific forces (P_o_) measured under oxygenated (95% O_2_ – 5% CO_2_) and hypoxic (95% N_2_-5% CO_2_) conditions. The maintained P_o_ is the percent ratio hypoxic to oxygenated P_o_.

* Greater than all other values, p<0.05.

### Study III: *In Vivo* Evaluation of SPO in a Rat Hind Limb Ischemia Model

**Figure 7:**
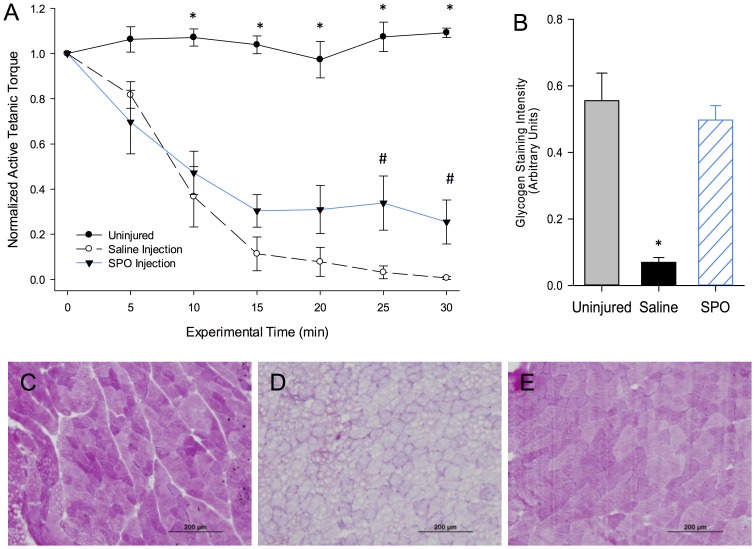
*In vivo* TA muscle contractility and glycogen depletion with and without SPO after 24 hours of hindlimb ischemia. (A) Ratio of isometric torque (Nmm) measured during repeated tetanic contractions (100 Hz pulse frequency, 0.1 ms pulse width, 200 ms train duration, 1 contraction every 5 minutes) normalized to initial torque values. Within a given time point, (*) Uninjured muscles maintained greater initial torque than all other groups and (#) SPO injected muscles maintained greater initial torque than Saline injected muscles (p<0.05). (B) Quantification of intramuscular glycogen (Periodic Acid Schiff stain) in TA muscles from Uninjured (C), Saline- (D), and SPO-injected (E) muscles. * Saline-injected muscles have significantly less intramuscular glycogen than all other groups (p<0.05). Values are means ± sem; sample size = 3/group.

Upon finding that SPO generated oxygen was capable of partially maintaining skeletal muscle resting metabolism under hypoxic conditions *in vitro (Study Two)*, we sought to determine the effectiveness of SPO for the preservation of ischemic skeletal muscle tissue *in vivo.* To promote ischemia in the rat hindlimb, the iliac artery was ligated for 24 hours, after which time the tibialis anterior muscle (the prime dorsiflexor) was treated and assessed for signs of tissue hypoxia. As a global metabolic index, TA muscle contractility was measured via neural stimulation of the common peroneal nerve in anesthetized rats (2.0–2.5%) for thirty minutes (contractions performed at 100 Hz, 200 ms, 5 minute resting interval) prior to harvesting muscles for glycogen content analysis. Over the 30-minute *in vivo* protocol, non-ligated control muscles did not demonstrate a reduction in torque and exhibited normal glycogen staining, indicating that with an intact blood supply, the contractile protocol does not induce fatigue (i.e., is not metabolically taxing) (Figure 7A). On the other hand, following iliac artery ligation, saline-injected TA muscles exhibited a ∼90% reduction in torque within 15 minutes, and out to 30 minutes, of contractile activity that corresponded with a nearly complete depletion of intramuscular glycogen (Figure 7A). These findings are consistent with others indicating increased susceptibility to muscular fatigue with hindlimb ischemia [38]. However, SPO injection maintained ∼30% of initial torque values out to 30 minutes of testing (Figure 7A; #p = 0.0443) and maintained glycogen staining to a similar level as non-ligated muscle (Figure 7B, C–E; p = 0.5687). These findings indicate that SPO-generated oxygen can, at least partially, maintain resting skeletal muscle metabolism *in vivo* in a model of partial ischemia.

## Discussion

A technology that delivers oxygen to hypoxic tissues in a fashion that is independent of vascular status (i.e., in either the absence of an intact vasculature or in the face of a compromised vascular supply) would represent an important medical advance with applications to tissue salvage, repair and regeneration following soft tissue trauma. This is the first report we are aware of that demonstrates that an oxygen-generating compound (SPO) ameliorates hypoxia-induced loss of skeletal muscle homeostasis and function both *in vitro* and *in vivo.* SPO was selected for this purpose because it theoretically produces sufficient amounts of oxygen to maintain skeletal muscle metabolism (Figure 1), via the catalytic decomposition of hydrogen peroxide. Herein we demonstrate, that a biologically and physiologically compatible concentration of SPO partially prevented: 1) the loss of contractility, HIF1α protein accumulation, glycogen depletion, and lipid peroxidation *in vitro* after a 30-minute incubation under hypoxic conditions (95% N_2_-5% CO_2_; 37°C) while muscles were relatively inactive (duty cycle <0.001); 2) the rise in baseline tension, indicative of a loss of intracellular Ca^2+^ homeostasis, *in vitro* during a contractile fatigue protocol (duty cycle = 0.1) performed in oxygenated conditions (95% O_2_ – 5% CO_2_; 37°C); and 3) the diminution of TA muscle torque and glycogen depletion in a partial hindlimb ischemia model *in vivo,* again while the muscles were relatively inactive (duty cycle <0.001). Further, we demonstrate the utility of providing an oxygen-generating compound (SPO), as compared to an oxygen-carrying compound (*see methods*), for preservation of skeletal muscle contractility following an acute bout of hypoxia *in vitro* (95% N_2_-5% CO_2_; 30 min.; 37°C; duty cycle <0.001), in the absence of an intact vasculature. Collectively, the results of these studies support our overarching hypothesis that SPO, an oxygen-generating compound, has the capacity to support resting skeletal muscle metabolism under otherwise hypoxic conditions.

In response to acute hypoxia, the loss of contractility in relatively inactive muscle results from an increased presence of free radicals and not the reduction of intramuscular high-energy phosphate content [21], [22], [23], [24], [25], [40], [42]. In this way, diminution of contractility may serve as a protective mechanism, disallowing hypoxic muscle from using limited energy stores for increased activity [23]. Previous reports have demonstrated that the loss of muscle contractility *in vitro* is partially prevented by attenuating elevations of reactive oxygen or nitrogen species during hypoxia [22], [25], [40] or by the return to oxygenated conditions following hypoxia ([40]; Also demonstrated in Supporting Figure S2). While there are many potential sites and modes for redox modification of proteins to modulate skeletal muscle contractility (e.g., alterations in excitation-contraction coupling vs. myofilament cross-bridge formation) [43], the reduction of myofilament maximal Ca^2+^ activated force (F_max_) mediated by reactive oxygen species and potentially peroxynitrite has been identified as playing a significant role in hypoxia-related loss of contractility [40], [42]. In the current study, we observed that SPO (1 mg/mL) significantly attenuated the rise in oxidative stress observed in hypoxic saline injected muscles, indicating that SPO-mediated maintenance of contractility appears to be due to an attenuation of hypoxia-driven alterations of the cellular redox status. Further, the downward and rightward shift in the force-frequency curve that we observed after hypoxia in saline injected muscle is suggestive of potentially multiple sites of contractile disruption (e.g., EC-uncoupling and reduced maximal activated force). Of note, SPO injection appeared to improve only the downward, but not the rightward shift of the force-frequency curve – consistent with previous findings of ROS and RNS (peroxynitrite) reducing F_max_ following hypoxia [40] or increased contractile activity under oxygenated conditions (95% O_2_) [44]. That the right-ward shift, indicative of EC-coupling disruption, was not affected by SPO treatment (Figure 3C) as compared to saline-injected muscles suggests that either a component of the EC-coupling machinery is more sensitive to hypoxia-driven perturbations to cellular homeostasis (e.g., [45], [46]) or that the injection itself is playing a role. Regardless, the findings of this study indicate that SPO conserves muscle contractility in hypoxic conditions by preventing related elevations in oxidative stress.

It is well recognized that isolated whole rat muscle preparations have the potential to develop a hypoxic core during *in vitro* studies [35], even in supra-oxygenated conditions (95% O_2_) [33], [34]. As muscle temperature and/or activity increase, the estimated radius of O_2_ diffusion from the bath media decreases exponentially [33], and consequently, contractility is diminished. Additionally, contractility may be depressed by increased oxidative stress in superficial fibers exposed to supra-physiological levels of O_2_ [44], as well as ion (e.g., K+) diffusion related alterations in membrane polarization [47]. Herein, a fatiguing contractile protocol, using a preparation designed to exacerbate the development of hypoxia within the muscle (*see methods*), was implemented to test the capacity of SPO (1 mg/mL) to ameliorate contractile dysfunction related to the development of a hypoxic core. In this setting, SPO did not alter the loss of active force (Figure 5A & B). However, elevations of resting tension during the active fatigue protocol were attenuated by SPO injection (Figure 5 C & D). Previous reports on whole muscle or isolated fibers have demonstrated that a rise in baseline tension with hypoxia (or anoxia) and with increased activity is the result of elevated resting cytosolic Ca^2+^ [22], [48]. Specifically, the rise in resting tension in hypoxic diaphragm results from extracellular Ca^2+^ influx secondary to plasmalemma or T-tubule membrane damage, which may be attenuated by the removal of Ca^2+^ from the bathing media or by a superoxide scavenger [22]. Thus, the ability of SPO to attenuate elevations of lipid peroxidation in hypoxic muscle (Figure 4), may in part explain the improved maintenance of resting tension during the contractile fatigue protocol. Based on previous reports, it is expected that the observed maintenance of baseline tension by SPO would have a concomitant improvement in active forces [22], [49]. As a potential explanation for this unexpected observation, we posit that accumulation of extracellular K^+^ within the core of the depolarized muscle fibers irrespective of treatment [47], rendered these fibers insensitive to the electrical stimulus (0.2 ms pulse width) used in this study, but that they were still capable of contributing to resting tension. These findings demonstrate that 1 mg/mL SPO can effectively preserve components of resting homeostasis when the muscle is under oxidative stress imparted by metabolic activity.

From a clinical standpoint, the utilization of SPO technology to provide an adequate source of oxygen for cells and tissues would be of extreme value in cases where oxygen diffusion limitations exacerbate tissue damage and restrict the spectrum of therapeutic possibilities. Clinical evidence suggests the potential for oxygen to increase the efficiency of salvage of ischemic skin flaps/grafts [50] and crush injuries [51], [52] in humans in studies using hyperbaric oxygen treatment. This has also been an effective method to reduce skeletal muscle ischemia/reperfusion injury in rats [53], [54]. To investigate the capacity of SPO to preserve skeletal muscle homeostasis *in vivo,* an optimized SPO concentration and dosage were correlated from *in vitro* results and applied in a partial hindlimb ischemia rat model. This model has previously been shown to reduce blood flow to the lower extremities at rest and during activity and to diminish muscle function out to fourteen days after ligation [38]. In this scenario, SPO injection mitigated the impact of ischemia-induced muscle tissue hypoxia after 24 hours of iliac artery ligation, as reflected by an improved maintenance of anterior crural muscle contractility and glycogen preservation, compared to saline injected groups (Figure 7A). Interestingly, the amount of SPO injected into the TA muscle provided theoretically only ∼22% of the oxygen required for resting skeletal muscle of this size (*see methods*). However, even this amount of SPO- derived supplemental O_2_ appeared capable of partially preserving muscle homeostasis in this model. More comprehensive animal models and analyses should be performed to further optimize concentrations, volumes and perhaps improved formulations of, SPO.

The primary advantage of an oxygen-generating versus oxygen-carrying material is the potential to deliver a sustained source of oxygen for the salvage of soft tissue following acute vascular trauma. The *in vitro* organ bath and *in vivo* studies performed herein validated the use of an oxygen-generating biomaterial, SPO, for the acute preservation of skeletal muscle viability in the absence of a functioning vasculature. Preceding the findings of the current study, SPO incorporated into scaffolds devoid of a vascular system has also been shown to improve cell survival and function of hypoxic 3T3 fibroblasts [55] and β cells and pancreatic islet cells [56]. In comparison, oxygen-carrying materials, such as PFCs, have improved skeletal muscle viability following ischemia, but these effects were observed with vascular delivery [57], [58]. To this end, the delivery of SPO for this indication (ischemic skeletal muscle secondary to vascular trauma/abalation) appears to be limited to intramuscular injection. While we demonstrate in the rat EDL muscle that bilateral needle injection results in acute reductions in contractility, it is not likely that this will present a significant limitation to translation to humans for the following reasons: 1) Intramuscular injection induces reparable damage to muscle fibers [59], which may result in an acute loss of contractility (as evidenced herein) but that would be far outweighed by the overall treatment outcome of greater soft tissue salvage; And, 2) intramuscular injection delivery in a matrix format that would allow for adequate diffusion of SPO through a large tissue bed is an accepted practice already utilized in clinical trials for other skeletal muscle therapies and indications [60], [61]. While the SPO injection protocol will clearly need to be further optimized in a large animal study, the present and preceding findings highlight oxygen generating materials as a potentially versatile system for providing required oxygen to cells and tissues under diverse conditions in which the vasculature is compromised or altogether absent.

The primary goal of these studies was to determine if SPO could preserve skeletal muscle homeostasis in the rat EDL or TA muscles when oxygen delivery was compromised. The improved maintenance of TA and/or EDL muscle contractility when these muscles were placed under hypoxic or ischemic conditions appears to be directly related to the provision of supplemental oxygen via SPO. Several lines of indirect evidence support this supposition: First, injection of the individual components of SPO (i.e., H_2_O_2_ & H_2_CO_3_) did not improve (or exacerbate the loss of) contractility following an *in vitro* hypoxia protocol, indicating that it is the specificity for the production of O_2_ via the catalytic decomposition of SPO that is required for tissue preservation in these studies. Second, glycogen levels were partially spared following hypoxic or ischemia testing when SPO, but not saline, was injected, suggesting that SPO-derived O_2_ was capable of supporting oxidative metabolism to at least some extent [1], [41]. And lastly, SPO attenuated hypoxia-induced HIF1α accumulation within myonuclei in EDL muscle, which is recognized as the prime regulatory factor in skeletal muscle mediated, hypoxia-induced gene expression [62]. Therefore, we posit that SPO at 1 mg/mL is a biologically and physiologically compatible oxygen-generating biomaterial that, at the very least, can partially support resting metabolism and thereby prevent redox modifications that characterize the acute response to hypoxia and may lead to otherwise irreversible damage in skeletal muscle. However, one should keep in mind that skeletal muscle has a broad metabolic range, wherein metabolic activity may rise ∼6–10 times that of resting metabolism during intense activity [20]. In addition, human muscles are comprised of mixed fibers types with a continuum of metabolic and functional phenotypes that may present added challenges to SPO treatment [63]. It is currently unclear what the metabolic requirements are *in vivo* for tissue salvage following limb polytrauma, although there are reports indicating that skeletal muscle metabolism is elevated in response to injury [19] or trauma [64]. In short, based on our initial observations with this novel material, in the current test systems, it is reasonable to conclude that SPO-mediated oxygen delivery (even with this first generation biomaterial formulation) would be adequate for preservation of muscle homeostasis and function following traumatic injuries that are expected to largely eliminate voluntary movement of the damaged/affected limb. Future studies will be aimed at directly testing this hypothesis, by assessing the capacity of SPO to provide supplemental oxygen for the preservation of oxidative metabolism in the context of extremity trauma.

## Conclusions

To the best of our knowledge, this is the first report that utilizes oxygen-generating compounds to document mitigation of a hypoxia-induced loss of muscle homeostasis both *in vitro* and *in vivo*. Most current comparable therapeutic devices and technologies utilize materials with enhanced oxygen carrying capacity, but are largely dependent on an intact vascular network. The major advantage of SPO, in the context of limb polytrauma in which limb perfusion is compromised, is that it is a self-generating source of oxygen in aqueous environments. The studies presented herein are a promising foundation on which further development of this technology will continue in order to fill a translational research gap in the area of muscle tissue salvage and restoration.

## Supporting Information

Figure S1 **Physiological compatibility of the delivery of SPO and its components to isolated EDL muscle.** (A) Effect of physical manipulations (needle) and vehicle (saline) injection on muscle contractility in oxygenated (95% O_2_–5%CO_2_) environment at 1, 60, 100 and 200 Hz stimulations. * Untreated muscle produced greater force at 100 and 200 Hz stimulations (p<0.05). (B) Comparison of SPO components (as in the 1 mg/mL dose of SPO) to saline and SPO injections. The individual components of 1.0 mg/mL SPO did not alter contractility as compared to either 1 mg/mL SPO or saline under oxygenated conditions. Values are means ± sem; sample sizes are listed in Table 2.
(TIF)

Figure S2 **Restoration of EDL muscle contractility upon reoxygenation following acute hypoxia.** EDL muscles underwent the hypoxic protocol testing described in Figure 3. At the end of the hypoxic period, the 95% N_2_- 5% CO_2_ gas was changed back to 95% O_2_–5% CO_2_ and contractility (200 Hz tetanic contraction) was assessed every 5 minutes for 15 minutes. In response to hypoxia, maximal force of the tissues was significantly decreased (black bar). Values are expressed as the maintenance of initial active force under oxygenated conditions. * Upon reoxygenation, there was a 15% increase in tetanic force (p<0.05). Values are means ± sem; sample size = 3.
(TIF)

Figure S3 **Improved maintenance of EDL muscle maximal tetanic force under hypoxic conditions.** Maximal tetanic force values (200 Hz, 200 ms train) measured at the end of hypoxic protocol listed in Figure 3 are expressed as the ratio of initial oxygenated maximal force. * In comparison to all other groups, the 1 mg/mL SPO injection maintained a greater fraction of initial force (p<0.05). Neither the 0.1 mg/mL SPO or the components (at concentrations equivalent to 1 mg/mL) of SPO, altered the maintenance of initial force compared to saline injected muscle. Values are means ± sem; sample sizes are listed in Table 2.
(TIF)
