# Supplementary figures and images for: Oxygen Generating Biomaterials Preserve Skeletal Muscle Homeostasis under Hypoxic and Ischemic Conditions
Source: PLoS One. 2013 Aug 26;8(8):e72485. doi: 10.1371/journal.pone.0072485 (PMC3753241; doi:10.1371/journal.pone.0072485)

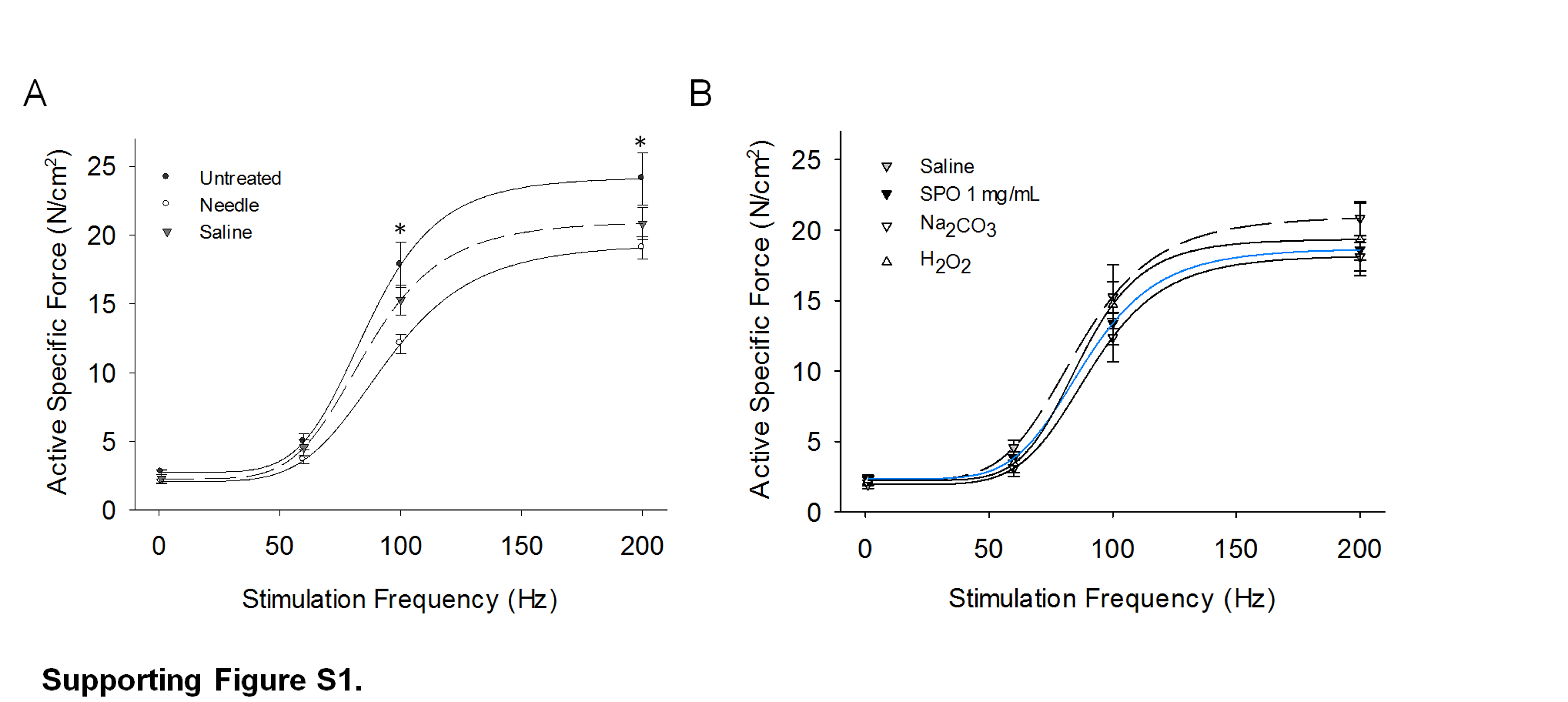

Supplement: Figure S1 — Physiological compatibility of the delivery of SPO and its components to isolated EDL muscle. (A) Effect of physical manipulations (needle) and vehicle (saline) injection on muscle contractility in oxygenated (95% O2–5%CO2) environment at 1, 60, 100 and 200 Hz stimulations. * Untreated muscle produced greater force at 100 and 200 Hz stimulations (p<0.05). (B) Comparison of SPO components (as in the 1 mg/mL dose of SPO) to saline and SPO injections. The individual components of 1.0 mg/mL SPO did not alter contractility as compared to either 1 mg/mL SPO or saline under oxygenated conditions. Values are means ± sem; sample sizes are listed in Table 2. (TIF) [file pone.0072485.s001.tif]

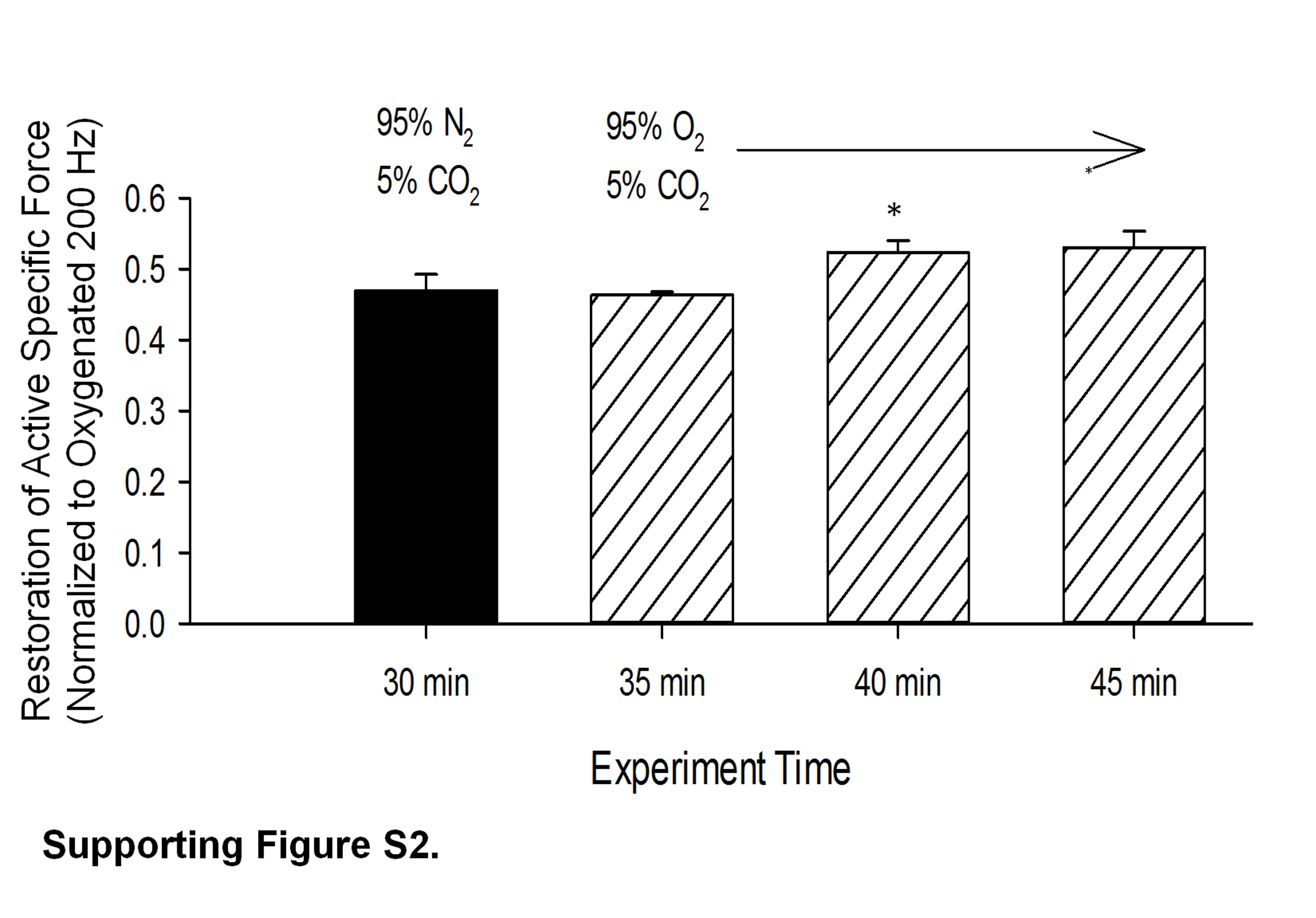

Supplement: Figure S2 — Restoration of EDL muscle contractility upon reoxygenation following acute hypoxia. EDL muscles underwent the hypoxic protocol testing described in Figure 3. At the end of the hypoxic period, the 95% N2- 5% CO2 gas was changed back to 95% O2–5% CO2 and contractility (200 Hz tetanic contraction) was assessed every 5 minutes for 15 minutes. In response to hypoxia, maximal force of the tissues was significantly decreased (black bar). Values are expressed as the maintenance of initial active force under oxygenated conditions. * Upon reoxygenation, there was a 15% increase in tetanic force (p<0.05). Values are means ± sem; sample size = 3. (TIF) [file pone.0072485.s002.tif]

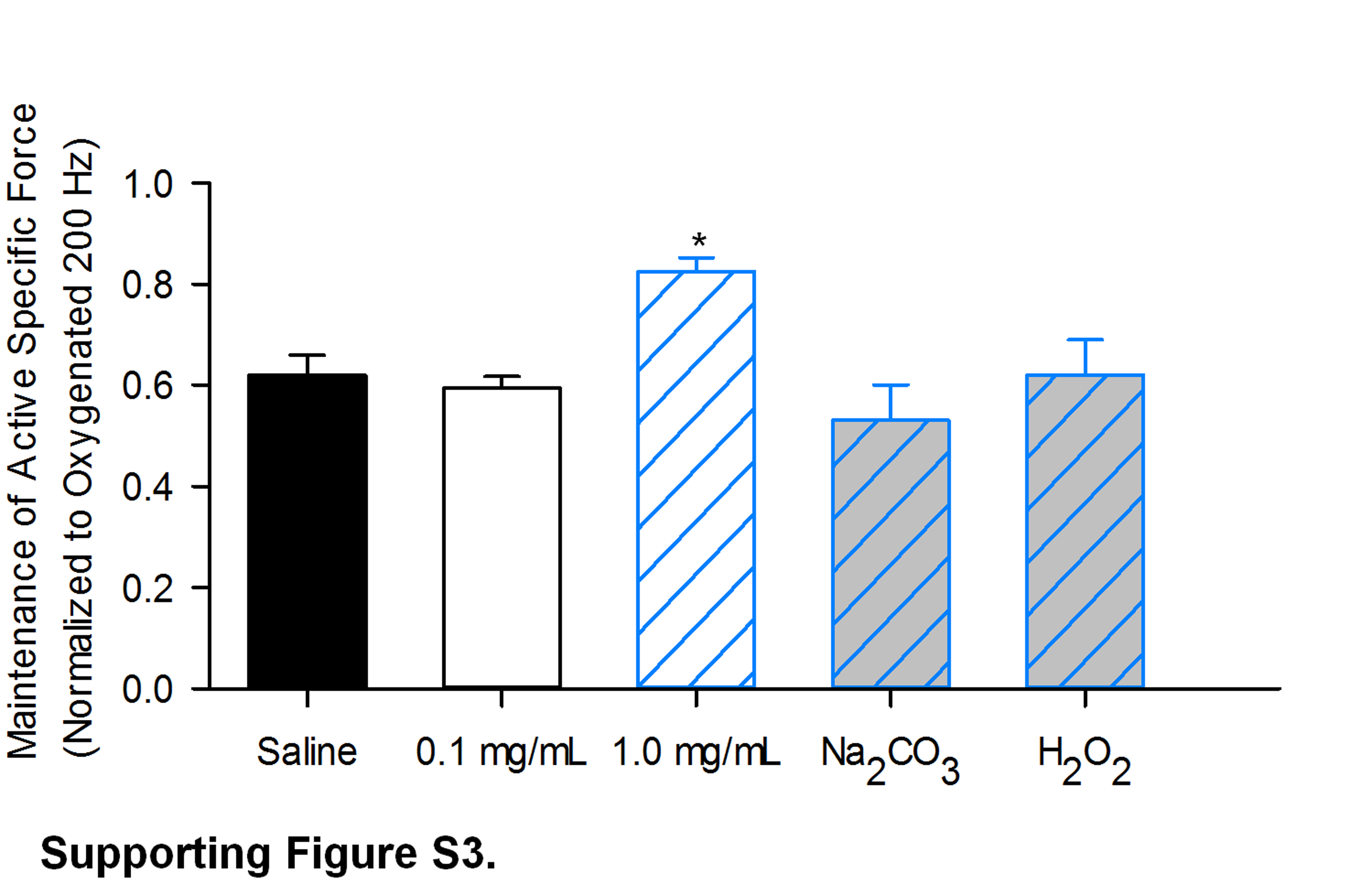

Supplement: Figure S3 — Improved maintenance of EDL muscle maximal tetanic force under hypoxic conditions. Maximal tetanic force values (200 Hz, 200 ms train) measured at the end of hypoxic protocol listed in Figure 3 are expressed as the ratio of initial oxygenated maximal force. * In comparison to all other groups, the 1 mg/mL SPO injection maintained a greater fraction of initial force (p<0.05). Neither the 0.1 mg/mL SPO or the components (at concentrations equivalent to 1 mg/mL) of SPO, altered the maintenance of initial force compared to saline injected muscle. Values are means ± sem; sample sizes are listed in Table 2. (TIF) [file pone.0072485.s003.tif]
